# Research progress of cancer cell membrane coated nanoparticles for the diagnosis and therapy of breast cancer

**DOI:** 10.3389/fonc.2023.1270407

**Published:** 2023-09-14

**Authors:** Zixia Zhou, Shengmin Zhang, Nianyu Xue

**Affiliations:** Department of Ultrasound Medicine, The First Affiliated Hospital of Ningbo University, Ningbo, China

**Keywords:** cancer cell membrane coated, nanoparticles, breast cancer, molecular imaging, theragnostic

## Abstract

Nanoparticles (NPs) disguised in the cell membrane are a new type of biomimetic platform. Due to their ability to simulate the unique biological functions of membrane-derived cells, they have become one of the hotspots of research at home and abroad. The tumor-specific antigen antibody carried by breast cancer cell membranes can modify nanoparticles to have homologous tumor targeting. Therefore, nanoparticles wrapped in cancer cell membranes have been widely used in research on the diagnosis and treatment of breast cancer. This article reviews the current situation, prospects, advantages and limitations of nanoparticles modified by cancer cell membranes in the treatment and diagnosis of breast cancer.

## 1 Introduction

In recent years, breast cancer has become the leading cause of cancer death in women (1). Early diagnosis and effective treatment of breast cancer are key to reducing mortality. However, traditional methods of diagnosing breast cancer often rely on tissue biopsy, which is limited by sampling errors and invasiveness. Moreover, there is a lack of effective diagnostic methods for detecting metastatic lesions of breast cancer. Additionally, the systemic side effects of breast cancer chemotherapy pose challenges to the survival of patients with underlying health conditions. In order to improve this problem, in recent years, nanoparticles drug delivery systems have been widely studied for targeted delivery of molecular probes/therapeutic drugs to achieve accurate early diagnosis and treatment of breast cancer. However, the lack of targeting of nanoparticles and the presence of problems such as tumor immune suppression have led to reduced treatment efficacy and tumor recurrence (2, 3). In recent years, some scholars have found that nanoparticles modified by cancer cell membranes have better immune evasion ability, permeability and targeting ability than unmasked nanoparticles in the treatment of breast cancer models, making it easier for therapeutic/diagnostic drugs to aggregate at the target location. This provides new possibilities for the precise diagnosis and chemical treatment of breast cancer (4). This article will review the progress of biomimetic nanoparticles wrapped in cancer cell membranes used for the diagnosis and treatment of breast cancer.

## 2 Cancer cell membrane coated nanoparticles and breast cancer

### 2.1 Breast cancer

Breast cancer originates from the glandular epithelial tissue of the breast and has high heterogeneity. Based on the expression of estrogen receptor, progesterone receptor, human epidermal growth factor receptor 2 and Ki-67, breast cancer can be divided into Luminal A type, Luminal B type, HER2-positive type and Basal-like type. The treatment and prognosis of breast cancer are closely related to its molecular typing. Currently, the basic treatment for breast cancer is surgery, radiotherapy and chemotherapy (5). However, there is still no completely effective treatment for breast cancer. Surgery is not suitable for every stage of cancer. Radiotherapy is harmful to normal tissues in the body and is not completely effective against cancer cells. Chemotherapy has certain limitations in the treatment of breast cancer. For example, the side effects of chemotherapy make it impossible for patients to receive further treatment. Secondly, cancer cells use drug transport pumps to remove drugs from inside the cells, resulting in distant metastasis that cannot be affected by chemotherapy drugs and tumor cells that are resistant to chemotherapy drugs (6).

### 2.2 Cancer cell membrane coated nanoparticles

**Figure 1:**
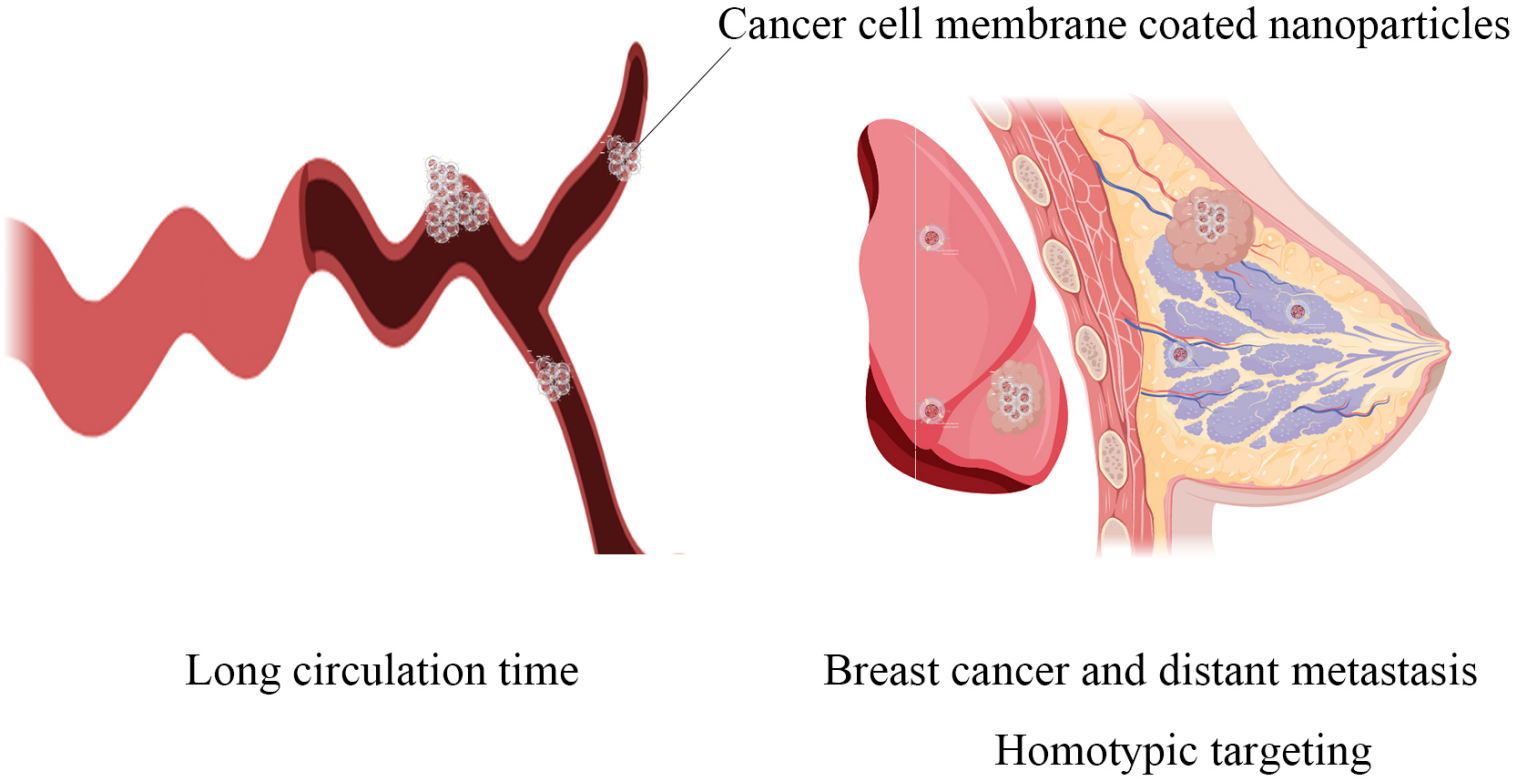
The main advantages of cancer cell-modifying nanoparticles: the significantly prolonged circulation time in the body and homotypic targeting towards both primary breast cancer lesions and distant metastatic lesions.

Cancer cell membrane coated nanoparticles (CCMNPs) are NPs coated with the lipid bilayer of cancer cell membranes. NPs are synthetic materials with stability and biocompatibility. NPs have been widely proven to be used to carry therapeutic or imaging drugs and enhance the stability of hydrophobic drugs, reduce toxicity and improve the efficiency of drugs on tumors (7). However, currently, most of the NPs drug delivery systems fail to meet clinical requirements in terms of circulation time *in vivo* and accumulation within tumor tissues. Therefore, in recent years, some scholars have proposed using cellular membrane to biologically modify NPs to evade the clearance by the immune system (8). Among the various cell membranes being extensively studied are red blood cell membrane, platelet membrane, stem cell membrane, immune cell membrane, extracellular vesicles and cancer cell membrane (9, 10). Compared to other cell membranes, cancer cell membrane has shown significant homologous targeting, with a much higher concentration in tumor tissues compared to nanoparticles encapsulated with other cell membranes. It also exhibits certain characteristics of prolonged blood circulation time, making it more promising in tumor treatment (11) (**Figure 1**). The cancer cell membrane coating has a variety of surface proteins derived from cancer cell membranes, including tumor-specific ligands, which exhibit biological complexity may be used for cancer vaccines and related applications (12). Using CCMNPs combines the advantages of synthetic materials and biological materials in a single biomimetic platform (13).

### 2.3 Preparation of cancer cell membrane coated nanoparticles

**Figure 2:**
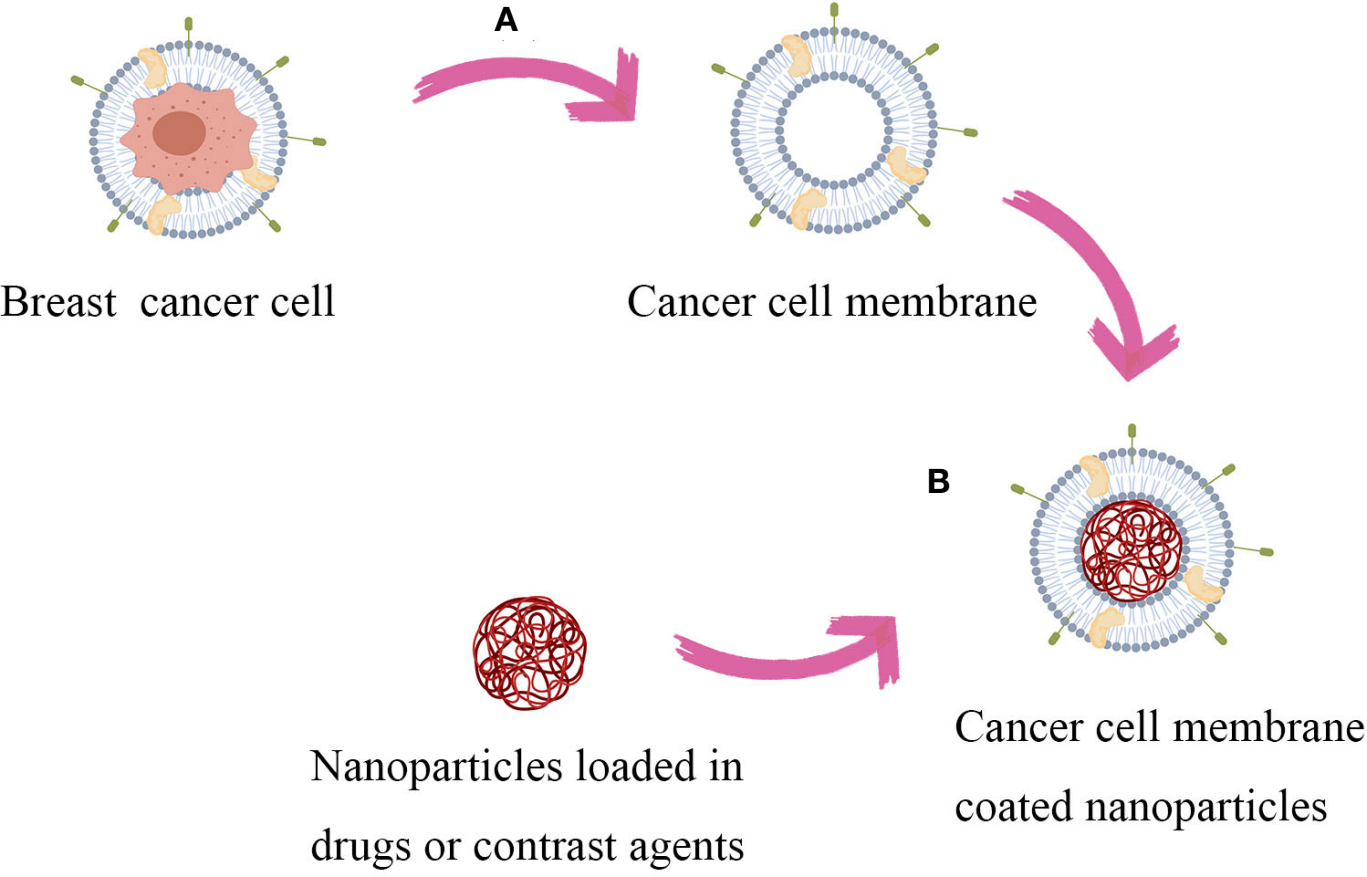
Synthesis process of CCMNPs: Firstly, obtain the target cancer cells and nanoparticles. **(A)** The cells are lysed and disrupted by utilizing a hypotonic solution treatment or repetitive freeze-thaw cycles. Continuous sucrose gradient centrifugation and ultrasonic washing are performed to remove cellular contents. Subsequently, the membrane-rich fraction is washed with a plasma buffer solution to obtain cellular membrane vesicles. **(B)** Fuse the obtained cancer cell membranes with nanoparticles using a mechanical extrusion/ultrasonic treatment/microfluidic electroporation-based method to obtain CCMNPs.

The brief process of synthesizing cancer cell membrane coated nanospheres is to obtain cancer cell membrane and nanoparticle core separately, followed by encapsulating the nanoparticle surface with the cancer cell membrane (**Figure 2**). The nanoparticles core of CCMNPs is mainly composed of poly-lactic acid-hydroxyethyl acrylate copolymer (PLGA), liposomes, ferric oxide, copper sulfide and up-conversion nanoparticles (14). This article mainly discusses the modification of cancer cell membranes and does not elaborate too much on the synthesis of nanoparticles. The integrity of functional proteins on the cell membrane surface is key to achieving its biomimetic camouflage. Therefore, the extraction of the cell membrane skeleton should be as gentle as possible to minimize possible deformation of membrane proteins. To obtain the required cancer cell membrane, cultured tumor cells are treated with low-permeability buffer or repeated freeze-thawing processes to mediate cell death. Subsequently, under the protection of protease inhibitors at 4 °C, the contents inside the cells, including nuclei, enzymes and other vesicles, are removed by discontinuous sucrose gradient centrifugation and ultrasonic washing, and finally the cell membrane can be collected (15). Then, the membrane-enriched fraction is washed with a plasma buffer to obtain cell membrane vesicles, which are further subjected to ultrasonic treatment and extruded through a porous polycarbonate membrane to obtain nanosized vesicles. Finally, the obtained cancer cell membrane is fused with nanoparticles to obtain CCMNPs. The commonly used methods for fusion include mechanical extrusion and ultrasonic treatment. Mechanical extrusion involves the use of a porous polycarbonate membrane to co-extrude the membrane and nanoparticles. By repeatedly pressing the nanoparticle-cell membrane mixture through polycarbonate membranes of different pore sizes, fusion between the nanoparticles and the cell membrane is achieved, taking advantage of the flowability of cell membrane vesicles (8). Ultrasonic treatment utilizes ultrasound-induced cavitation bubbles to disrupt the membrane structure, allowing the membrane to reassemble around the nanoparticles. In terms of cost and efficiency for large-scale production, ultrasonic treatment is more favorable. However, specific adjustments in ultrasound power, frequency, and duration are required to minimize denaturation of surface proteins on the cell membrane (16). Despite these efforts, both methods still have relatively low efficiency and success rates, posing a significant bottleneck for the further application of cancer cell membrane-modified nanoparticles. Some scholars have used microfluidic electroporation-based method to achieve complete membrane coverage on the core nanoparticles, during which process pulse voltage, duration, and flow velocity should be optimized (17).

## 3 The application in breast cancer imaging and diagnosis

### 3.1 Cancer cell membrane coated fluorescent molecular probe imaging

Upconversion nanoparticles (UCNPs) convert near-infrared radiation (NIR) into visible light, and are a new generation of fluorescent probes with broad application prospects (18, 19). UCNPs have photochemical characteristics, such as significant light penetration depth, narrow emission peak, excellent photostability, low toxicity and no background fluorescence, which are especially suitable for imaging of superficial organ cancers such as breast cancer (20). RAO et al. assembled MDA-MB-435 human breast cancer cell membrane-modified β-NaYF_4_:Er^3+^, Yb^3+^UCNs (CC-UCNs), and also assembled cell membranes from melanoma/prostate/squamous/cell/colorectal cells, and synthesized corresponding CC-UCNs. The homologous binding of CC-UCNs was studied *in vitro* by flow cytometry and confocal microscopy. Significant binding was observed when the cell membrane of CC-UCNs matched the cancer cell type, while almost no targeting was displayed when mismatched. When CC-UCNs derived from MDA-MB-435 cells was injected into mice implanted with homologous tumors, their homologous tumor grafts showed obvious Upconversion luminescence, and accumulated higher than CC-UCNs from other cell lines (13, 21). Another study by CHEN et al. showed that CCM molecular probe successfully reduced the interception of liver and kidney on it, the cell adhesion molecules on the surface of camouflaged NPs had homologous targeting binding, achieved high aggregation of tumor, and could perform real-time high spatial resolution imaging (22).

### 3.2 Cancer cell membrane coated paramagnetic nanoparticles in MRI imaging

Currently, in clinical practice, X-ray mammography, ultrasound, and MRI imaging are mainly used for breast cancer diagnosis. Compared to the other two, MRI is considered to have better soft tissue high spatial resolution. And due to the relative popularity of its equipment, the development of MRI imaging using nanoparticles with cancer cell membrane coating is believed to be easier in clinical practice than single molecular probe imaging. The research on MRI targeted imaging using molecular probes combined with paramagnetic materials has a long history. It has been considered more valuable in disease diagnosis due to its high r1 and r2 values and long blood circulation time (23). However, the combination of molecular probes and paramagnetic materials alone has always been unsatisfactory in terms of biocompatibility and *in vivo* circulation time. In recent years, based on this, it is believed that there is potential for better blood circulation time and biocompatibility by attaching cancer cell membrane coating on the surface of nanoparticles (24). According to some reports, MRI contrast agents using molecular probes coated with cancer cell membranes combined with paramagnetic materials have shown better aggregation effects in cancer models such as lung cancer. The use of cancer cell membrane coated nanoparticles in MR-enhanced imaging has achieved better imaging results (25, 26).

In breast cancer research, Zhang et al. demonstrates that paramagnetic substances coated with breast cancer cell membranes exhibit enrichment within cancer cells and possess potential for *in vivo* applications (27). some scholars have synthesized 4T1 cancer cell membrane coated Poly lactic-co-glycolic acid-Prussian blue (PLGA-PBs) materials. In a mouse homogenous tumor model, it has been demonstrated that in T1-weighted imaging, there is a clear boundary between the tumor tissue and surrounding normal tissue, with clear anatomical structure. In T1-weighted quantitative analysis, the enhancement rate of the cancer cell-modified group (159.632 ± 8.549%) is significantly higher than that of the molecular material without cancer cell membrane coated (76.784 ± 3.346%). This indicates the evident homogenous targeting effect of breast cancer cell membrane modification and its enrichment in cancer tissue (28).

Other scholars have also used MDA-MB-231 cancer cell membranes coated NaGdF_4_:Yb,Tm@NaGdF_4_(UNNPs)to perform MR imaging on both a mouse model with homogenous breast cancer and a mouse model with MCF-7 breast cancer. The results indicate that compared to the group directly using UCNPs, the group injected with cancer cell membrane coated UCNPs shows a more pronounced enhancement in the tumor area. Furthermore, the enhancement in the tumor area is more pronounced in the group injected into the MDA-MB-231 tumor compared to the MCF-7 group. This suggests that cancer cell-modified nanoparticles have a certain degree of discriminatory ability for different molecular subtypes of breast cancer, which cannot be achieved by traditional MRI imaging methods (29).

### 3.3 Cancer cell membrane coated ultrasound microbubble imaging

The use of ultrasound scattering from microbubbles to achieve contrast-enhanced ultrasound (CEUS) has attracted much attention for its potential in cancer molecular imaging (30). Currently, micrometer-sized gas bubble ultrasound contrast agents have been widely used in clinical practice. In order to more effectively distinguish between malignant and benign lesions, nanoscale particles modified with cancer cell membranes that have the ability to target extravascular targets are introduced. Recently, JUGNIOT et al. developed cancer cell membrane-modified nanobubbles with targeting ability for ultrasound contrast imaging of triple-negative breast cancer. Their shell is composed of TNBC MDA-MB-231 cell membrane and added surfactant to reduce surface tension, and filled with octafluoropropane gas inside. The experimental results showed that the nanoparticles had specific adhesion effect *in vitro* model and showed good homologous affinity, which was conducive to the aggregation of CCMNBs in the tumor. In mouse model CEUS imaging, CCMNBs showed affinity for different subtypes of breast cancer models, and compared with non-targeted NBs, the peak intensity and area under the curve of time intensity map showed that CCMNBs signal was significantly enhanced (2.1 times, P=0.004 and 3.6 times, P=0.009, respectively), and had higher stability and longer retention period. In terms of biosafety, no residue was found in all tissues 24h after injection, and no biological damage was caused. Immunofluorescence analysis further confirmed the presence of CCMNBs in the tumor microenvironment. It showed that CCMNBs can break through the limitation of universal cancer biomarker recognition, make tumor necrosis factor biomarker targeting not affected by the high heterogeneity of breast cancer tissue, and thus improve the diagnostic ability and potential drug targeting delivery ability (31).

Compared to other imaging methods, using CEUS for targeted imaging is more convenient and cost-effective, and is more conducive to clinical application. Therefore, it has broad application prospects. In addition, despite this, there are still few studies on CEUS imaging with cancer cell membrane coated nanoparticles, which is believed to be a focus worthy of observation in the future.

## 4 Application in breast cancer therapy

### 4.1 Cancer cell membrane coated nanoparticle in chemotherapy

Chemotherapy is an important method for breast cancer treatment, especially for triple-negative breast cancer (TNBC) and advanced breast cancer patients who have lost surgical indications, which can effectively relieve symptoms, improve patient quality of life and prolong survival. In recent years, with the development of nanotechnology, to solve the problems of low bioavailability, poor efficiency and large side effects of traditional chemotherapy drug delivery methods, people have studied the use of nanoparticles to encapsulate chemotherapy drugs for delivery, but there are still problems of insufficient body circulation time and easy clearance by the immune system (32, 33). In one study, SUN et al. developed a CCMNPs for the treatment of breast cancer with distant metastasis. Its core is composed of nanoparticles polymerized by poly(caprolactone) (PCL) and pluronic copolymer F68 loaded with paclitaxel (PTX), and the outer layer is a coating formed by 4T1 breast cancer cell membrane. Thomsen-Friedenreich antigen(TF-antigen), epithelial cell calcium adhesion protein (E-cadherin) and CD47 were further confirmed to be related to breast cancer cell adhesion and recognition. The experimental results showed that using this CCMNPs-DDS can selectively make PTX highly accumulate in the primary tumor and lung tissue metastatic lesions, and showed significant anti-tumor and anti-metastasis effects (34).

Besides, the use of cancer cell membrane to wrap poly (lactic acid) (PLA), spiky-shell mC@SiO2 nanomotor, nanomicelle lactose-DOX (lac-DOX) loaded chemotherapy drugs have proved the high targeting, high accumulation characteristics of CCMNPs in breast cancer treatment, and have good biosafety (35, 36).

In addition, some scholars have modified nanoparticles loaded with chemotherapy drugs with hybrid membranes formed by mixing cancer cell membranes with other cell membranes, such as cancer cell membrane-macrophage membrane, cancer cell membrane-red blood cell membrane (37, 38). GONG’s cancer cell membrane-macrophage membrane, which combines the advantages of cancer cell membrane and macrophage, showed stronger inflammation driving ability and targeting specific metastasis ability, and significantly improved multi-targeting ability of metastatic lesions in breast cancer lung metastasis model. It provides a promising biomimetic nano-platform for effective treatment of breast cancer metastasis.

### 4.2 Cancer cell membrane coated nanoparticle optical therapy

Optical therapy mainly includes photodynamic therapy (PDT) and photothermal therapy (PTT), which is a therapy that uses photosensitizer (PS) to be excited by light of a specific wavelength to release reactive oxygen or heat to achieve the purpose of treating cancer (39). Since PS shows high accumulation level in the near-nuclear regions such as mitochondria, endoplasmic reticulum, etc. of most breast cancer cells, PDT and PTT are considered to have broad prospects for the treatment of breast cancer (40–42). Using CCMNPs for targeted delivery of PS can effectively increase the local target area blood drug concentration, and reduce the drug accumulation in other tissues and organs, and reduce side effects. CHENG et al. reported CCM wrapped dendritic large pore mesoporous silica nanoparticles loaded with copper sulfide and R484 (CCM@DLMSN@CuS/R484) for PTT treatment of TNBC. The results showed that CCM@DLMSN@CuS/R484 had high TNBC targeting ability, and induced effective photothermal ablation of primary TNBC tumors under 980nm laser irradiation. Therefore, tumor antigen produced by responding to photothermal effect and gradually released R484, combined with AUNP-12 separated from tumor cells CCM@DLMSN@CuS/R484 synergistically exerted tumor vaccine inoculation and T lymphocyte activation function in weak acidic tumor microenvironment, promoted immune remodeling, and prevented TNBC recurrence and metastasis (43).

In addition, recently WU et al. developed a core-shell nano-platform (HM/D-I-BL), using hollow mesoporous manganese dioxide (HM) to coat biomimetic cancer cell membrane, for tumor photodynamic therapy. Cell uptake and fluorescence imaging studies confirmed that HM/D-I-BL can be accurately delivered to the tumor site. HM/D-I-BL has the characteristics of *in situ* O2 generation in the tumor after laser irradiation, followed by enhanced chemotherapy/phototherapy, indicating its role as a TME-responsive nano-enzyme in alleviating tumor hypoxia in the presence of H2O2. In addition, HM/D-I-BL showed good fluorescence and magnetic resonance imaging properties, which provided reliable multimodal image-guided combined tumor treatment for future precise treatment (44).

Recently, some nano systems that utilize photosensitizers to boost cuproptosis and proton pump inhibitors (PPIs) to enhance PDT have shown promising therapeutic effects for breast cancer and its distant metastases. It has been proven that these systems exhibit improved efficacy when modified with certain biomembranes, highlighting their potential for further development (45–47). Currently, there is still a lack of relevant experiments on cancer cell membrane modifications, which may be a crucial focus for future supplementation.

### 4.3 Immune targeting therapy using cancer cell membrane coated

Some recent studies have shown that molecular targeted therapy, such as epidermal growth factor receptor tyrosine kinase inhibitor (EGFR-TKI), has great potential for the treatment of triple-negative breast cancer. However, clinical data showed that EGFR-TKI drugs such as afatinib failed to achieve the expected (48). Therefore, a novel drug delivery method that can improve the efficacy of the drug is needed. WANG et al. used tumor cell membrane to wrap AFT/2-BP@PLGA@MD nanoparticles to significantly enhance the ability of AFT to inhibit tumor cell proliferation and migration *in vitro*. In addition, the nanoparticles showed enhanced tumor targeting ability *in vivo*, significantly inhibited the growth and metastasis of 4T1 tumors, and prolonged the survival of tumor-bearing mice. The nanoparticles also elicited anti-tumor immune response. It showed a novel and effective strategy for the treatment of refractory TNBC (49).

In addition to delivering immunotherapeutic drugs, the specific antigens present on the cancer cell membrane are believed to be able to train the immune system as a tumor vaccine. Although conceptually attractive, the clinical efficacy has been unsatisfactory due to the similarity between cancer tissue and healthy tissue. Therefore, some scholars have proposed using adjuvants that enhance immune stimulation by encapsulating the cancer cell membrane to promote effective antigen presentation and activation of downstream immune processes. KROLL et al. used CpG oligodeoxynucleotide 1826 (CpG), a nucleic acid-based immune adjuvant known to trigger antigen-presenting cell maturation, encapsulated in PLGA and coated on B16-F10 mouse melanoma cells. Experimental results using a syngeneic tumor mouse model showed that this nanovaccine formulation achieved significant control of tumor growth in a therapeutic setting (12). Similarly, other scholars have achieved promising results by using B16-F10 mouse melanoma cell membrane encapsulation with different immune-stimulating adjuvants (50, 51). In breast cancer research, RAO and other scholars utilized genetically edited cancer cell membrane coated magnetic nanoparticles(gCM-MNs) overexpressing SIRPα variants. In the 4T1 breast cancer cell mouse model, gCM-MNs exhibited significant control over local tumor growth and distant tumor metastasis, resulting in a significant extension of overall mouse survival. The combination of cell membrane coating nanotechnology and gene editing technology provides a safe and robust new strategy for activating the body’s immune response against cancer (52).

Currently, there is still limited research on cancer cell membrane-modified nanoparticle immunotherapy specifically targeting breast cancer models. Immunotherapy remains a hot topic in tumor research, and further studies are eagerly anticipated.

## 5 Safety and future clinical applications of cell membrane coated nanoparticles

Currently, in animal experimental studies, no direct harm to animal models has been observed with CCMNPs. Additionally, due to their relatively high accumulation within tumors, CCMNPs often reduce the side effects caused by therapeutic drugs. However, it is worth noting that CCMNPs have a longer circulation time in the body compared to the individual use of drugs or nanoparticle encapsulation, which undoubtedly increases safety risks. On the other hand, existing experiments still lack long-term observations of experimental animals and their offspring, thus the long-term risks of CCMNPs application cannot be ruled out.

Furthermore, although some metal, inorganic, and polymer nanoparticles have been approved for human use or are in advanced clinical trials (53), the use of biological cell membrane encapsulation in CCMNPs may classify the resulting drugs as biologics. This raises higher requirements for the heterogeneity and batch-to-batch variability of CCMNPs in different patients. This still requires further work from scientists and engineers.

## 6 Concluding remarks and future perspective

Cancer cell membrane-modified nanoparticles show better ability to evade immune surveillance, permeability and targeting. Homologous targeting delivery of imaging agents and therapeutic agents, precise imaging of tumors, destruction of tumor cells in primary and metastatic lesions, and induction of immune response are the main applications of cancer cell membrane-modified nanoparticles in breast cancer diagnosis and treatment, and have the potential to become one of the important drug delivery methods for related diagnosis and treatment in the future. There is still limited research on breast cancer-related experiments, such as breast cancer contrast-enhanced ultrasound and immunotherapy, which requires further in-depth research. Furthermore, the main problems that still need to be solved are the unclear mechanism of cancer cell homologous targeting, the unstable properties of cancer cell membrane-modified nanoparticle synthesis, how to achieve stable large-scale production, and the lack of experimental data on humans. Successfully addressing these challenges will enable this novel type of nanoparticle to benefit patients widely and achieve personalized precision medicine.

## Author contributions

ZZ: Writing – original draft. SZ: Funding acquisition, Writing – review & editing. NX: Writing – review & editing.

## References

[1] Sung H Ferlay J Siegel RL Laversanne M Soerjomataram I Jemal A. Global cancer statistics 2020: GLOBOCAN estimates of incidence and mortality worldwide for 36 cancers in 185 countries. CA Cancer J Clin (2021) 71:209–49. doi: 10.3322/caac.21660 33538338

[2] Kawiak A. Molecular research and treatment of breast cancer. Int J Mol Sci (2022) 23. doi: 10.1038/s41467-018-03705-y PMC9455640 36077013

[3] Wang Y Li S Wang X Chen Q He Z Luo C. Smart transformable nanomedicines for cancer therapy. Biomaterials (2021) 271:120737. doi: 10.1016/j.biomaterials.2021.120737 33690103

[4] Wang H Liu Y He R Xu D Zang J Weeranoppanant N. Cell membrane biomimetic nanoparticles for inflammation and cancer targeting in drug delivery. Biomater Sci (2020) 8:552–68. doi: 10.1039/C9BM01392J 31769765

[5] Burstein HJ Curigliano G Thürlimann B Weber WP Poortmans P Regan MM. Customizing local and systemic therapies for women with early breast cancer: the St. Gallen International Consensus Guidelines for treatment of early breast cancer 2021. Ann Oncol Off J Eur Soc Med Oncol (2021) 32:1216–35. doi: 10.1016/j.annonc.2021.06.023 PMC9906308 34242744

[6] Prieto-Vila M Takahashi RU Usuba W Kohama I Ochiya T. Drug resistance driven by cancer stem cells and their niche. Int J Mol Sci (2017) 18(12):2574. doi: 10.3390/ijms18122574 PMC5751177 29194401

[7] Allahverdiyev AM Parlar E Dinparvar S Bagirova M Abamor ES. Current aspects in treatment of breast cancer based of nanodrug delivery systems and future prospects. Artif Cells Nanomed Biotechnol (2018) 46:S755–62. doi: 10.1080/21691401.2018.1511573 30260234

[8] Kroll AV Fang RH Zhang L. Biointerfacing and applications of cell membrane-coated nanoparticles. Bioconjugate Chem (2017) 28:23–32. doi: 10.1021/acs.bioconjchem.6b00569 PMC5471317 27798829

[9] Liu C Wang Y Li L He D Chi J Li Q. Engineered extracellular vesicles and their mimetics for cancer immunotherapy. J Control Release (2022) 349:679–98. doi: 10.1016/j.jconrel.2022.05.062 35878728

[10] Fan Z Jiang C Wang Y Wang K Marsh J Zhang D. Engineered extracellular vesicles as intelligent nanosystems for next-generation nanomedicine. Nanoscale horizons (2022) 7:682–714. doi: 10.1039/D2NH00070A 35662310

[11] Fang RH Hu CM Luk BT Gao W Copp JA Tai Y. Cancer cell membrane-coated nanoparticles for anticancer vaccination and drug delivery. Nano Lett (2014) 14:2181–8. doi: 10.1021/nl500618u PMC3985711 24673373

[12] Kroll AV Fang RH Jiang Y Zhou J Wei X Yu CL. Nanoparticulate delivery of cancer cell membrane elicits multiantigenic antitumor immunity. Adv Mater (2017) 29(47). doi: 10.1002/adma.201703969 PMC5794340 29239517

[13] Jin J Bhujwalla ZM. Biomimetic nanoparticles camouflaged in cancer cell membranes and their applications in cancer theranostics. Front Oncol (2019) 9:1560. doi: 10.3389/fonc.2019.01560 32039028 PMC6985278

[14] Wang R Zhang Z Liu B Xue J Liu F Tang T. Strategies for the design of nanoparticles: starting with long-circulating nanoparticles, from lab to clinic. Biomater Sci (2021) 9:3621–37. doi: 10.1039/D0BM02221G 34008587

[15] Gao W Zhang L. Coating nanoparticles with cell membranes for targeted drug delivery. J Drug Target (2015) 23:619–26. doi: 10.3109/1061186X.2015.1052074 26453159

[16] Hu CM Zhang L Aryal S Cheung C Fang RH Zhang L. Erythrocyte membrane-camouflaged polymeric nanoparticles as a biomimetic delivery platform. Proc Natl Acad Sci U.S.A. (2011) 108:10980–5. doi: 10.1073/pnas.1106634108 PMC3131364 21690347

[17] Rao L Cai B Bu L-L Liao Q-Q Guo S-S Zhao X-Z. Microfluidic electroporation-facilitated synthesis of erythrocyte membrane-coated magnetic nanoparticles for enhanced imaging-guided cancer therapy. ACS Nano (2017) 11:3496–505. doi: 10.1021/acsnano.7b00133 28272874

[18] Wang Y Feng M Lin B Peng X Wang Z Lv R. MET-targeted NIR II luminescence diagnosis and up-conversion guided photodynamic therapy for triple-negative breast cancer based on a lanthanide nanoprobe. Nanoscale (2021) 13:18125–33. doi: 10.1039/D1NR05847A 34605506

[19] Zhu D Lyu M Huang Q Suo M Liu Y Jiang W. Stellate plasmonic exosomes for penetrative targeting tumor NIR-II thermo-radiotherapy. ACS Appl Mater Interfaces (2020) 12:36928–37. doi: 10.1021/acsami.0c09969 32814380

[20] Qin Z Li Y Gu N. Progress in applications of pRussian blue nanoparticles in biomedicine. Advanced healthcare materials (2018) 7:e1800347. doi: 10.1002/adhm.201800347 29974662

[21] Rao L Bu LL Cai B Xu JH Li A Zhang WF. Cancer cell membrane-coated upconversion nanoprobes for highly specific tumor imaging. Adv Mater (2016) 28:3460–6. doi: 10.1002/adma.201506086 26970518

[22] Chen Z Zhao P Luo Z Zheng M Tian H Gong P. Cancer cell membrane-biomimetic nanoparticles for homologous-targeting dual-modal imaging and photothermal therapy. ACS Nano (2016) 10:10049–57. doi: 10.1021/acsnano.6b04695 27934074

[23] Liu C Gao Z Zeng J Hou Y Fang F Li Y. Magnetic/upconversion fluorescent NaGdF4:Yb,Er nanoparticle-based dual-modal molecular probes for imaging tiny tumors in vivo. ACS Nano (2013) 7:7227–40. doi: 10.1021/nn4030898 23879437

[24] Zhai Y Su J Ran W Zhang P Yin Q Zhang Z. Preparation and application of cell membrane-camouflaged nanoparticles for cancer therapy. Theranostics (2017) 7:2575–92. doi: 10.7150/thno.20118 PMC5558554 28819448

[25] Meng X Wang J Zhou J Tian Q Qie B Zhou G. Tumor cell membrane-based peptide delivery system targeting the tumor microenvironment for cancer immunotherapy and diagnosis. Acta Biomater (2021) 127:266–75. doi: 10.1016/j.actbio.2021.03.056 33813091

[26] Zhao Y Pan Y Zou K Lan Z Cheng G Mai Q. Biomimetic manganese-based theranostic nanoplatform for cancer multimodal imaging and twofold immunotherapy. Bioact Mater (2023) 19:237–50. doi: 10.1016/j.bioactmat.2022.04.011 PMC9048124 35510176

[27] Zhang D Ye Z Wei L Luo H Xiao L. Cell membrane-coated porphyrin metal-organic frameworks for cancer cell targeting and O(2)-evolving photodynamic therapy. ACS Appl Mater Interfaces (2019) 11:39594–602. doi: 10.1021/acsami.9b14084 31577410

[28] Chen Q Zhang L Li L Tan M Liu W Liu S. Cancer cell membrane-coated nanoparticles for bimodal imaging-guided photothermal therapy and docetaxel-enhanced immunotherapy against cancer. J Nanobiotechnology (2021) 19:449. doi: 10.1186/s12951-021-01202-x 34952587 PMC8710014

[29] Fang H Li M Liu Q Gai Y Yuan L Wang S. Ultra-sensitive nanoprobe modified with tumor cell membrane for UCL/MRI/PET multimodality precise imaging of triple-negative breast cancer. Nanomicro Lett (2020) 12:62. doi: 10.1007/s40820-020-0396-4 34138297 PMC7770711

[30] Versluis M Stride E Lajoinie G Dollet B Segers T. Ultrasound contrast agent modeling: A review. Ultrasound Med Biol (2020) 46:2117–44. doi: 10.1016/j.ultrasmedbio.2020.04.014 32546411

[31] Jugniot N Massoud TF Dahl JJ Paulmurugan R. Biomimetic nanobubbles for triple-negative breast cancer targeted ultrasound molecular imaging. J Nanobiotechnology (2022) 20:267. doi: 10.1186/s12951-022-01484-9 35689262 PMC9185914

[32] Babu A Templeton AK Munshi A Ramesh R. Nanodrug delivery systems: a promising technology for detection, diagnosis, and treatment of cancer. AAPS PharmSciTech (2014) 15:709–21. doi: 10.1208/s12249-014-0089-8 PMC4037475 24550101

[33] Xu X Ho W Zhang X Bertrand N Farokhzad O. Cancer nanomedicine: from targeted delivery to combination therapy. Trends Mol Med (2015) 21:223–32. doi: 10.1016/j.molmed.2015.01.001 PMC4385479 25656384

[34] Sun H Su J Meng Q Yin Q Chen L Gu W. Cancer-cell-biomimetic nanoparticles for targeted therapy of homotypic tumors. Adv Mater (2016) 28:9581–8. doi: 10.1002/adma.201602173 27628433

[35] Han L Xu Y Guo X Yuan C Mu D Xiao Y. Cancer cell membrane-coated biomimetic platform for targeted therapy of breast cancer in an orthotopic mouse model. J Biomater Sci Polym Ed (2020) 31:1538–51. doi: 10.1080/09205063.2020.1764163 32362234

[36] Zhou M Xing Y Li X Du X Xu T Zhang X. Cancer cell membrane camouflaged semi-yolk@Spiky-shell nanomotor for enhanced cell adhesion and synergistic therapy. Small (2020) 16:e2003834. doi: 10.1002/smll.202003834 32877017

[37] Sun M Duan Y Ma Y Zhang Q. Cancer cell-erythrocyte hybrid membrane coated gold nanocages for near infrared light-activated photothermal/radio/chemotherapy of breast cancer. Int J Nanomedicine (2020) 15:6749–60. doi: 10.2147/IJN.S266405 PMC7494427 32982231

[38] Gong C Yu X You B Wu Y Wang R Han L. Macrophage-cancer hybrid membrane-coated nanoparticles for targeting lung metastasis in breast cancer therapy. J Nanobiotechnology (2020) 18:92. doi: 10.1186/s12951-020-00649-8 32546174 PMC7298843

[39] Li X Lovell JF Yoon J Chen X. Clinical development and potential of photothermal and photodynamic therapies for cancer. Nat Rev Clin Oncol (2020) 17:657–74. doi: 10.1038/s41571-020-0410-2 32699309

[40] Hammerer F Poyer F Fourmois L Chen S Garcia G Teulade-Fichou MP. Mitochondria-targeted cationic porphyrin-triphenylamine hybrids for enhanced two-photon photodynamic therapy. Bioorg Med Chem (2018) 26:107–18. doi: 10.1016/j.bmc.2017.11.024 29174053

[41] Leandro FZ Martins J Fontes AM Tedesco AC. Evaluation of theranostic nanocarriers for near-infrared imaging and photodynamic therapy on human prostate cancer cells. Colloids Surf B Biointerfaces (2017) 154:341–9. doi: 10.1016/j.colsurfb.2017.03.042 28365423

[42] Chen T Luo X Zhu LW Xiang JF Fang CF Zhu DM. Biomimetic single-atom nanozyme system for efficient inhibition of gastric cancer ascites *via* SO2 gas-enhanced nanocatalytic cancer therapy. Chem Eng. J (2023) 467:10. doi: 10.1016/j.cej.2023.143386

[43] Cheng Y Chen Q Guo Z Li M Yang X Wan G. An intelligent biomimetic nanoplatform for holistic treatment of metastatic triple-negative breast cancer *via* photothermal ablation and immune remodeling. ACS Nano (2020) 14:15161–81. doi: 10.1021/acsnano.0c05392 33143424

[44] Wu M Chen T Wang L Akakuru OU Ma X Xu J. The strategy of precise targeting and in *situ* oxygenating for enhanced triple-negative breast cancer chemophototherapy. Nanoscale (2022). doi: 10.21203/rs.3.rs-1300836/v1 35635070

[45] Ning S Lyu M Zhu D Lam JWY Huang Q Zhang T. Type-I AIE photosensitizer loaded biomimetic system boosting cuproptosis to inhibit breast cancer metastasis and rechallenge. ACS Nano (2023) 17:10206–17. doi: 10.1021/acsnano.3c00326 37183977

[46] Zhu D Ling R Chen H Lyu M Qian H Wu K. Biomimetic copper single-atom nanozyme system for self-enhanced nanocatalytic tumor therapy. Nano Res (2022) 15:7320–8. doi: 10.1007/s12274-022-4359-6

[47] Zhu D Zhang T Li Y Huang C Suo M Xia L. Tumor-derived exosomes co-delivering aggregation-induced emission luminogens and proton pump inhibitors for tumor glutamine starvation therapy and enhanced type-I photodynamic therapy. Biomaterials (2022) 283:121462. doi: 10.1016/j.biomaterials.2022.121462 35272223

[48] Vasudevan S Adejumobi IA Alkhatib H Roy Chowdhury S Stefansky S Rubinstein AM. Drug-induced resistance and phenotypic switch in triple-negative breast cancer can be controlled *via* resolution and targeting of individualized signaling signatures. Cancers (Basel) (2021) 13(19):5009. doi: 10.3390/cancers13195009 PMC8507629 34638492

[49] Wang X Zhu X Li B Wei X Chen Y Zhang Y. Intelligent biomimetic nanoplatform for systemic treatment of metastatic triple-negative breast cancer *via* enhanced EGFR-targeted therapy and immunotherapy. ACS Appl Mater Interfaces (2022). doi: 10.1021/acsami.2c02925 35549005

[50] Jiang Y Krishnan N Zhou J Chekuri S Wei X Kroll AV. Engineered cell-membrane-coated nanoparticles directly present tumor antigens to promote anticancer immunity. Adv Mater (2020) 32:e2001808. doi: 10.1002/adma.202001808 32538494 PMC7669572

[51] Yang R Xu J Xu L Sun X Chen Q Zhao Y. Cancer cell membrane-coated adjuvant nanoparticles with mannose modification for effective anticancer vaccination. ACS Nano (2018) 12:5121–9. doi: 10.1021/acsnano.7b09041 29771487

[52] Rao L Zhao SK Wen C Tian R Lin L Cai B. Activating macrophage-mediated cancer immunotherapy by genetically edited nanoparticles. Adv Mater (2020) 32:e2004853. doi: 10.1002/adma.202004853 33089578 PMC7686299

[53] Anselmo AC Mitragotri S. Nanoparticles in the clinic: An update. Bioengineering Trans Med (2019) 4:e10143. doi: 10.1002/btm2.10143 PMC6764803 31572799

